# Methane transport and emissions from soil as affected by water table and vascular plants

**DOI:** 10.1186/1472-6785-13-32

**Published:** 2013-09-08

**Authors:** Gurbir S Bhullar, Majid Iravani, Peter J Edwards, Harry Olde Venterink

**Affiliations:** 1 Institute of Integrative Biology, Plant Ecology, ETH Zurich, Universitätstrasse 16, Zurich 8092, Switzerland; 2 Research Institute of Organic Agriculture (FiBL), Ackerstrasse 21, Postfach 219, Frick 5070, Switzerland; 3 Department of Natural Resources, Isfahan University of Technology, Isfahan 84156, Iran; 4 Plant Biology and Nature Management, Vrije Universiteit Brussel, Pleinlaan 2, Brussels 1050, Belgium

**Keywords:** CH_4_, Chimney, Climate change, Greenhouse gas, Plant species, Transport, Wetlands

## Abstract

**Background:**

The important greenhouse gas (GHG) methane is produced naturally in anaerobic wetland soils. By affecting the production, oxidation and transport of methane to the atmosphere, plants have a major influence upon the quantities emitted by wetlands. Different species and functional plant groups have been shown to affect these processes differently, but our knowledge about how these effects are influenced by abiotic factors such as water regime and temperature remains limited. Here we present a mesocosm experiment comparing eight plant species for their effects on internal transport and overall emissions of methane under contrasting hydrological conditions. To quantify how much methane was transported internally through plants (the chimney effect), we blocked diffusion from the soil surface with an agar seal.

**Results:**

We found that graminoids caused higher methane emissions than forbs, although the emissions from mesocosms with different species were either lower than or comparable to those from control mesocosms with no plant (i.e. bare soil). Species with a relatively greater root volume and a larger biomass exhibited a larger chimney effect, though overall methane emissions were negatively related to plant biomass. Emissions were also reduced by lowering the water table.

**Conclusions:**

We conclude that plant species (and functional groups) vary in the degree to which they transport methane to the atmosphere. However, a plant with a high capacity to transport methane does not necessarily emit more methane, as it may also cause more rhizosphere oxidation of methane. A shift in plant species composition from graminoids to forbs and/or from low to high productive species may lead to reduction of methane emissions.

## Background

Wetlands are the largest natural source of the important greenhouse gas methane (CH_4_), contributing one-third to global emissions [1]. The gas is generated under anoxic conditions by methanogenic microbes (Archaea) [2,3], but the amounts reaching the atmosphere are affected by abiotic factors including temperature, pH, nutrients and water table [2,4-9]. Plants also influence the amounts of CH_4_ emitted from wetlands in various ways. They may enhance emissions, both by providing a carbon substrate for methanogenesis in the form of root exudates [10,11], and by transporting CH_4_ internally from the rhizosphere to the atmosphere [12-15]; and they may reduce emissions, by creating oxidising conditions in the rhizosphere [14,16]. The relative importance of these processes varies among plant species [4,11,17-21]; while many studies have found CH_4_ fluxes to the atmosphere to be increased by the presence of vascular plants [11,22,23], others have found them to be decreased [7,24-26]. These contradictory results may partly be related to the conditions under which the studies were performed, with factors such as water table also playing a role [27,28].

Moisture conditions are known to have a large effect upon CH_4_ emissions from soils. In arctic coastal plains, Morrissey & Livingston [29] found that CH_4_ emissions from inundated sites were 12 times higher than from sites where the water table was 5 cm below soil surface. Similarly, Moore and Dalva [30] found a negative logarithmic relationship between CH_4_ emissions and depth of the water table. These results are not unexpected, since a low water-table depth is associated with more oxidising conditions [7], and hence with lower emissions from unsaturated soils [30-32]. However, plants can transport CH_4_ produced in the rhizosphere through their roots, stems and leaves, thereby by-passing the upper oxic soil layer. Indeed, there have been many studies showing that more than half of CH_4_ emitted from wetland soils, including rice paddies, was transported internally by plants [13,20,33-36]; and in a study of Alaskan tundra vegetation, 92–98% of CH_4_ was attributed to plant-mediated transport [29]. There are also clear differences in the importance of the ‘chimney effect’ among plant functional types, including trees, grasses and forbs [37-40]. In a clipping experiment, for example, Ding et al. [20] found that cyperaceous plants have a higher capacity to transport CH_4_ (73-86% of total emissions) than graminaceous plants (28-31% of total emissions). To understand the importance of this mechanism for CH_4_ emissions from wetlands, it is necessary to measure the chimney effect for a range of wetland species under conditions of both high and low water table. Furthermore, to avoid possible confounding effects upon emissions of processes such as root exudation [11], these measurements be made using a substrate that is not carbon limited. To the best of our knowledge, no such studies have yet been carried out.

Finding a relationship between CH_4_ emissions and functional plant types under varying plant growth conditions is an important task, as it would be useful for modelling CH_4_ fluxes from various vegetation zones and for designing future mitigation strategies. The interactions among plants and abiotic factors affecting CH_4_ emissions may also differ among functional plant groups [11,41] and therefore merit further study.

Many studies have reported a positive relationship between plant productivity and CH_4_ emissions from soil [23,42], which was attributed to increased root exudation and gas exchange rates [6,21]. However, some workers have found either a negative or no relationship between productivity of vegetation and CH_4_ emission [11,26,43]. Hence, more work will be needed before we are able to predict the effects of altered productivity in wetlands, for instance through eutrophication or climate change.

We conducted a mesocosm experiment using eight plant species of European wetlands, including both forbs and graminoids. Our main aim was to investigate how CH_4_ emissions from soils are influenced by interactions of plant species (and functional plant groups) with depth of water table. Our specific hypotheses were:

i. Vascular plants increase CH_4_ emissions to the atmosphere in proportion to their capacity for plant-mediated transport.

ii. Graminoids cause higher CH_4_ emissions than forbs.

iii. The effect of plant species on CH_4_ emissions from wetlands varies according to the depth of the water table.

iv. Methane emissions are negatively related to plant biomass.

## Methods

The experiment was conducted in a greenhouse in Zurich, Switzerland during April-September 2009. We used eight species characteristic of European wetlands, including four forbs (*Caltha palustris* L., *Mentha aquatica* L., *Lycopus europaeus* L., *Rumex hydrolapathum* Huds*.*) and four graminoids (*Anthoxanthum odoratum* L., *Carex rostrata* Stokes, *Eriophorum angustifolium* Honckeny, *Glyceria maxima* (Hartm.) Holmb.). The plants were grown from seed that was either collected in the field in north-eastern Switzerland, or purchased from a company specialized in wild plants (Die Wildstaudengärtenerei, Eschenbach, Switzerland).

To minimise effects due to the diverse origins of the plants, we separated individual seedlings, washed their roots carefully, and planted them in small pots containing sand. After four weeks, small, uniform plants were transplanted in 2.5 litre (Diameter 120 mm & Height 230 mm) plastic mesocosms containing soil. The water table in the mesocosms could be monitored by means of a transparent tube that was connected to the mesocosms at the bottom. We conducted preliminary tests on a number of different soils with the aim of choosing a substrate that was capable of producing CH_4_; in particular, we looked for a soil that was not carbon limited, so as to minimise possible differences between species due to root exudation of organic compounds. Based on these tests we selected for the experiment an organic-rich soil that we purchased in a garden shop (Bio-Universalerde, ökohum gmbh).

The experiment was planted with 14 replicates of each species and the control (bare soil). Any plants that died within the first two weeks of the experiment were replaced. During the initial stages of the experiment, the soil in the mesocosms was kept inundated by irrigating daily with deionised water and keeping the water table at the soil surface level. To ensure uniform growth and development, all plants were provided with uniform conditions for first 2.5 months.

After 2.5 months, in mid-July, the water level in half of the replicate mesocosms was allowed to fall through evapotranspiration to 5 cm below the soil surface and was maintained at this level for rest of the experiment; in the remaining mesocosms the water level was maintained at the soil surface. Thus, the experiment consisted of eight plant species and one ‘no-plant control’; supplied with two levels of water treatment and replicated 7 times.

Methane was measured using a Photo Acoustic Field Gas-Monitor type 1412 (Innova AirTech Instruments Ballerup, Denmark) fitted with a moisture filter to dry the air before analysis. The instrument was calibrated with a gas chromatograph and yielded very consistent results [17]. All mesocosms were sampled for CH_4_ emissions during first half of August 2009. Each mesocosm was incubated in a transparent Plexiglas chamber for 20 minutes and the change in CH_4_ concentration inside the chamber during this time was recorded.

After an initial measurement of the gas flux with the mesocosm unsealed (f_us_), the soil surface was sealed with a viscous agar solution (1% w/v), described in detail elsewhere [44]. The agar solidified within a few minutes, and effectively blocked most gas exchange across the soil surface. Directly after sealing, the mesocosms were placed back in the air-cleaned chamber, and CH_4_ emission during the next 20 minutes was recorded (f_s_). The chimney effect (i.e. transport through plants) was calculated as: Chimney (%) = (f_s_ / f_us_)*100. The room temperature in which the measurements were made was around 25°C, and we used the corresponding density of CH_4_ (0.656 mg/cc) to calculate the CH_4_ emitted from each mesocosm. Since we can not rule out that sealing the soil with agar increased the relative fluxes of methane that were emitted through the plants (instead of through the soil), we note that this method shows the potential for the plants to act as conduits but possibly not the real importance in the actual soil-plant system.

After the gas flux measurement, the agar seal was removed and the plants were harvested. Aboveground and belowground biomass were measured after drying at 70°C for 48 hours. Root volume was measured using the pycnometer method as described by Jensen et al. [45], and root density was calculated by dividing the dry root mass by root volume.

**Figure 1:**
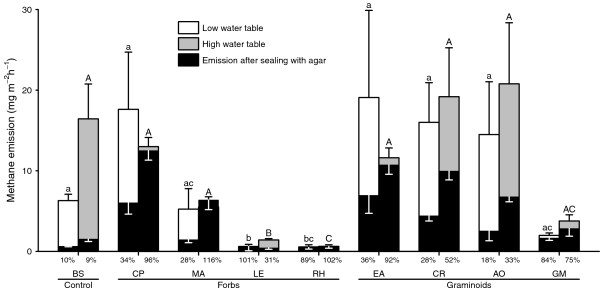
**Mean CH**_**4 **_**emission rates from peat mesocosms with different plant species, under unsealed conditions and after sealing the soil surface with agar.** Black error marks on top show standard error for unsealed emissions and white error marks inside the bars show standard error for sealed emissions. Small and capital letters above the error bars indicate significant differences in CH_4_ emission rates relative to mesocosms with bare soil only, for low water and high water treatments, respectively. Note that all (white, grey and black) bars start at zero, these are not cumulative bars. High water table means the soil was kept saturated up to surface level throughout the experiment; under the low water table treatment, water table was kept constant at five centimetres below the soil surface. Percent values below each bar indicate the chimney effect caused by each species under that particular treatment i.e. the proportion of total emission being transported via plant. BS: Bare soil (non-plant control), CP: Caltha palustris, MA: *Mentha aquatica*, LE: *Lycopus europaeus*, RH: *Rumex hydrolapathum*, AO: *Anthoxanthum odoratum*, CR: *Carex rostrata*, EA: *Eriophorum angustifolium*, GM: Glyceria maxima. Graph shows the original data while the significant differences are based on the statistical analysis of log-transformed data.

**Figure 2:**
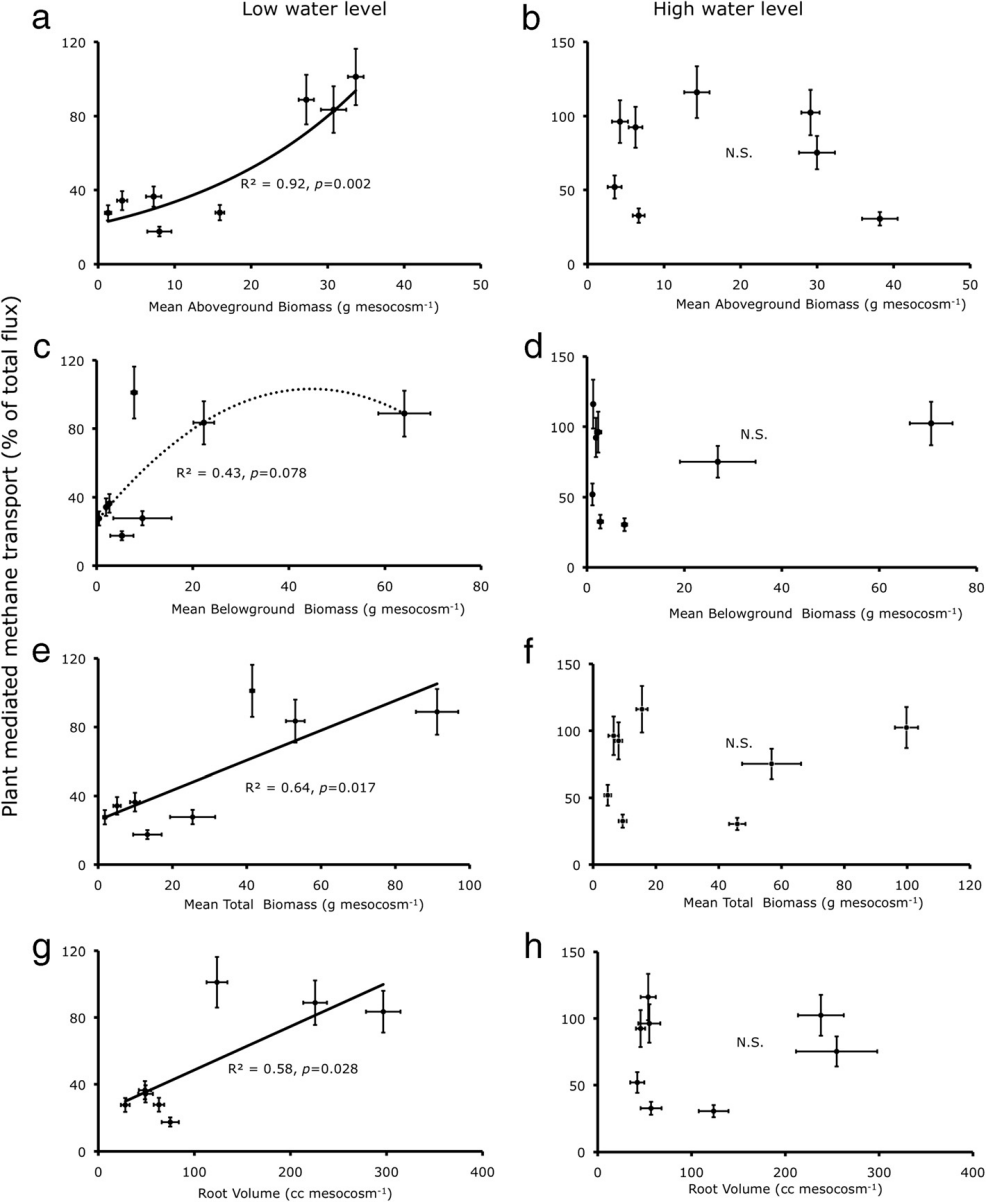
**Proportion of CH**_**4 **_**flux transported through the plant (chimney %) per species plotted against plant biomass and root characteristics, (a,c,e,g) under the low water level (i.e. 5 cm below soil surface); and (b,d,f,h) under high water level (at soil surface).** We tested linear, quadratic and inverse regressions and the solid lines were drawn if relationship was significant (*p*<0.05), dotted line for tendency (0.05≤*p*<0.1) and no line for insignificant relationship (*p*≥0.1). Note the scale differences of x-axis.

**Figure 3:**
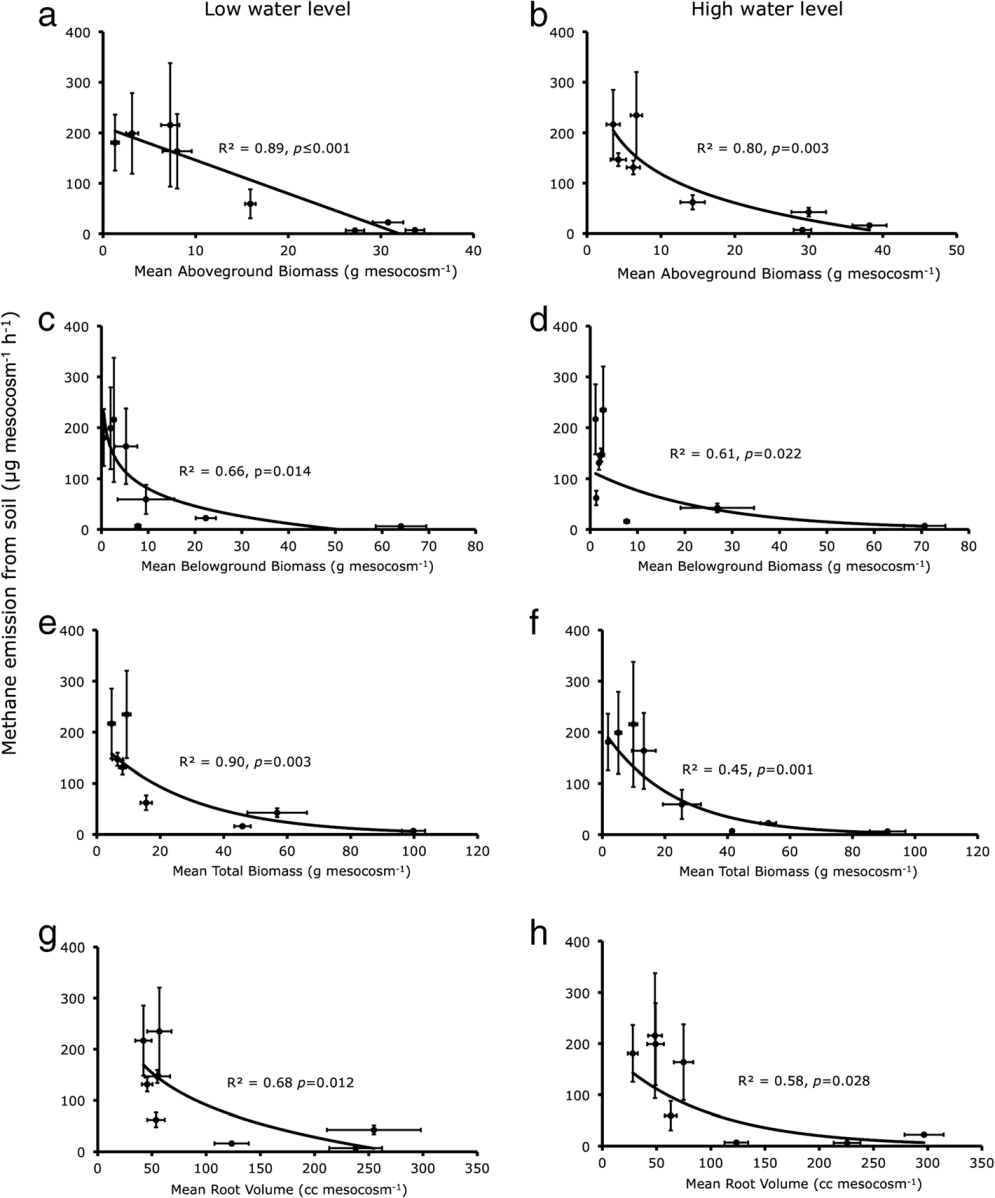
**Mean CH**_**4 **_**emission from the soil in presence of different species plotted against plant biomass and root characteristics, (a,c,e,g) under the low water level (i.e. 5 cm below soil surface); and (b,d,f,h) under high water level (at soil surface).** We tested linear, and exponential regressions and the solid lines were drawn if relationship was significant (*p*<0.05), dotted line for tendency (0.05≤*p*<0.1) and no line for insignificant relationship (*p*≥0.1). Note the scale differences of the x-axis.

**Figure 4:**
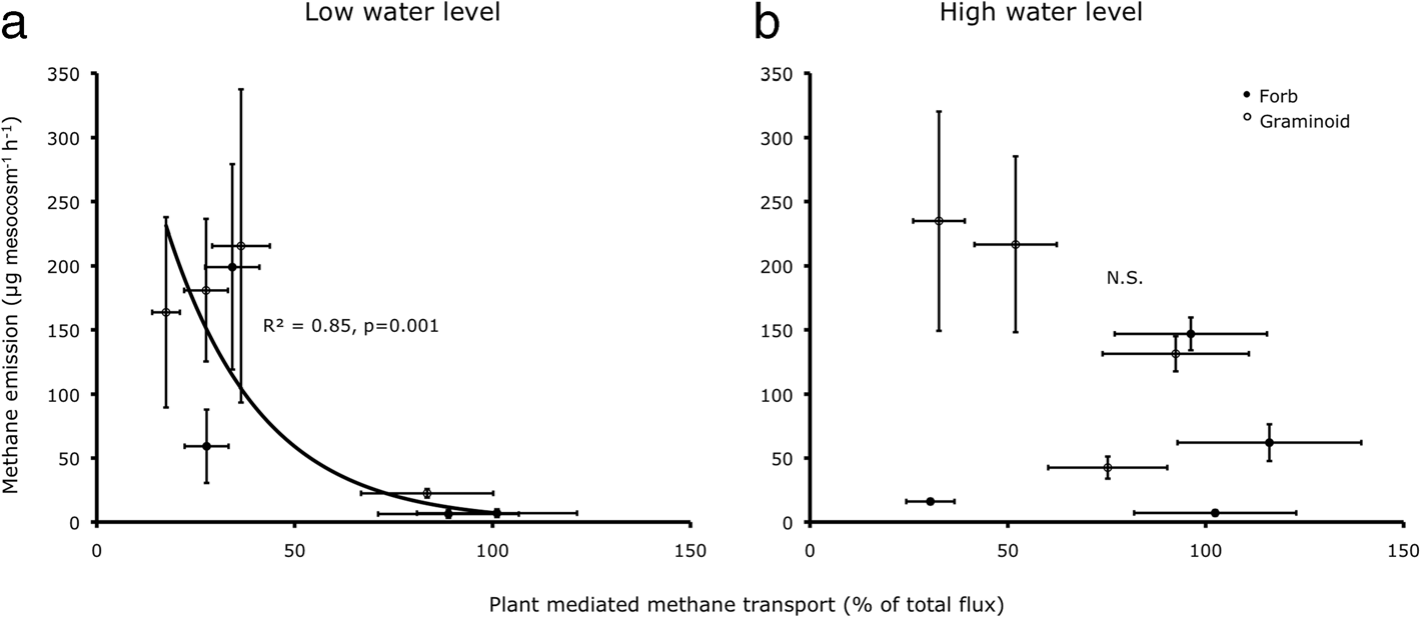
**Mean CH**_**4 **_**emission per species *versus *mean CH**_**4 **_**flux transported by plant (chimney %) per species under (a) the low water level (i.e. 5 cm below soil surface) and (b) the high water level (at soil surface).**

The effects of the fixed factors (i.e. plant species and water table) on CH_4_ emissions were tested using ANOVA. The same method was used to test for the interactions between plant species, water table and sealing treatments. If the main plant species effect was significant, ‘Dunnett’s multiple comparison test’ (family-wise significance level of *a*=0.05) was performed to test for differences in CH_4_ emissions and/or chimney effects among plant species and the bare soil control. A similar procedure was used to test for the interactions between plant functional groups (i.e. grasses and forbs) and the other factors. We applied paired-sample t-tests to check for differences in CH_4_ emissions between unsealed and sealed conditions for each species and also differences in CH_4_ emissions between functional groups (*a*=0.05). Regression analysis was used to test for any correlation between CH_4_ emission rates (and ‘% Chimney’) and various plant parameters, both with average values per species and for all mesocosms independent of species. Prior to data analysis, data on the CH_4_ emissions were subjected to log transformation to meet the criteria of normality and homogeneity. All analyses were performed in R release 2.8.1 (R Development Core Team, 2008). Untransformed data are presented in the figures. In Figure 1, we have used the standard units of mg m^-2^ hr^-1^. However, in Figures 2, 3, 4, we have intentionally used the original units in which the data was obtained i.e. μg mesocosm^-1^ hr^-1^, in order to avoid the error in regression analysis that might get introduced while converting biomass data from per mesocosm to per meter square. If required, CH_4_ emission rates can be converted from μg hr^-1^mesocosm^-1^ to mg hr^-1^ m^-2^ by multiplying with a factor of 0.088.

## Results

The flux of CH_4_ from the soil varied according to the plant species (Table 1, Figure 1). In contrast to the expected increase in the presence of a plant, CH_4_ emissions from all the species were either lower than or not significantly different from the bare soil control mesocosms (Figure 1). *Lycopus europaeus*, and *Rumex hydrolapathum* (both forbs) significantly reduced CH_4_ emissions in comparison to bare soil control, and *Mentha aquatica* (forb) and *Glyceria maxima* (graminoid) also reduced emissions, but not significantly (Figure 1). Graminoids and forbs differed significantly (F_1,14_=5.7, *p*=0.03) in their effect on overall CH_4_ emissions, with graminoids causing higher emissions than forbs.

**Table 1:** **Effect of species and water table on CH**_**4 **_**emission from soil (Degrees of freedom, F values and significance of 2-way ANOVA)**

**Source**	**Df**	**F**
Species	8	24.6***
Water Level	1	13.3***
Species* Water	8	1.2

P≤0.05; **P≤0.01; ***P≤0.001.

Lowering the water table caused a significant reduction in CH_4_ emissions from almost all mesocosms, including the control (Table 1, Figure 1), but there was no significant interaction between water table and plant species (Table 1). Some species, like *Caltha palustris, Eriophorum angustifolium, Carex rostrata* and *Anthoxanthum odoratum* tended to cause higher emissions at either low or high water table, but the differences were not significant (Figure 1).

Sealing of the mesocosms resulted in reduced CH_4_ emissions in all cases, the average reduction across all species being 36% (Figure 1). Nevertheless, upon sealing some plant species (*Caltha palustris, Eriophorum angustifolium, Carex rostrata*, and *Anthoxanthum odoratum*) caused a higher CH_4_ emission than that from the sealed bare soil, indicating that these plants were acting as chimneys (Figure 1). However, there was no significant interaction between ‘sealing’, water table and plant species (*F*_8, 216_ = 0.82, *p*=0.58) that would point to a stronger ‘chimney’ effect under either high or low water table (Figure 1).

The ‘percent chimney effect’ (i.e. the proportion of CH_4_ flux being transported through the plant) was directly proportional to the average aboveground biomass, total biomass and root volume of the plant under the low water treatment, but not under the high water treatment (Figure 2a-h). Furthermore, the overall CH_4_ emissions were negatively related to biomass (Figure 3).

## Discussion

Contrary to several studies in which plants were found to increase CH_4_ emissions, we found that mesocosms with plants emitted similar or even lower amounts of CH_4_ than those with bare soil [11,13,22,40,46,47]. Although, some species (e.g. *Anthoxanthum odoratum, Carex palustris, Eriophorum angustifolium* and *Carex rostrata*) tended to produce higher emissions, these were not statistically different from the bare soil. This indicates that in a carbon-rich soil such as we used in this experiment, any extra carbon from root exudation was insufficient to cause a significant increase in emissions. Therefore, most of the plant effects observed here were likely due to either rhizosphere oxidation or internal transport of CH_4_ (i.e. the chimney effect). As intended, the higher availability of labile carbon in the soil enabled us to rule out one mechanism (i.e. the increase in CH_4_ emissions due to root exudation by plants) and so focus on the other two mechanisms.

Methane emissions from three species (*Lycopus europaeus*, *Rumex hydrolapathum* and *Glyceria maxima*) were around 70%-95% lower than those from bare soil. These reductions, which were evident in both water level treatments and in sealed and unsealed mesocosms, were most probably due to oxidation of CH_4_ in the rhizosphere (Figure 1). Similar results have been reported in other studies [14,16,24-26,48]. Indeed, rhizosphere oxidation has been shown to account for reduction of between 20 and 97% of all CH_4_ produced in the soil [3,26,49,50], with considerable differences among species. For example, CH_4_ emission from rice monocultures were twice as high as those from mixtures with rice and weeds (*Lipocarpha sp., Rotala indica* and *Ludwigia epilobiodes*; [14,25], probably because of more rhizosphere oxidation in the mixtures.

In a previous experiment, graminoids were found to transport significantly more CH_4_ internally than forbs [44]. This could partly explain why in the experiment described here, graminoids caused higher CH_4_ emissions than forbs. However, we also found that species with a higher chimney effect also possess a greater capability to reduce emissions, presumably by transporting oxygen to the rhizosphere (Figure 4; discussed below). Thus, the effect of a plant species upon overall CH_4_ fluxes cannot be gauged from its capacity to transport CH_4_ internally, alone. Nevertheless, our results suggest that the two functional plant groups, graminoids and forbs, differ in their influence upon CH_4_ emissions from wetland soil. To understand the underlying mechanisms for these differences among functional plant groups, further investigations would be required, perhaps with larger sets of plant species and groupings based on different functional traits such as root characteristics and aerenchyma formation. It has been suggested that characterising vegetation in terms of plant functional traits could provide a simple and effective method for predicting CH_4_ emissions [43,51-53]. Such an approach would be useful for designing efficient mitigation and management strategies for future.

Lowering the water table, reduced CH_4_ emissions from the control mesocosms as well as from those containing a plant. This is in accordance with other studies, showing that CH_4_ emissions are greatly reduced when the surface soil is aerobic [7,30-32]. And despite the absence of any interaction between water table and overall CH_4_ emissions (Table 1), there were interesting differences between species in how the water-level treatment affected the proportion of CH_4_ transported internally. It seems that lowering the water table by 5 cm in this organic soil was not enough to produce a significant interaction at the species level. Grunfeld and Brix [32] found that a difference of 8 cm in water depth had a major effect upon the methanogenic activity of sandy soils but not of organic soils, presumably because of the higher water holding capacity of the latter. Besides this, the plant species tended to behave differently with respect to the chimney effect under two water levels (see percent values below bars in Figure 1). Most species (*C. palustris*, *M. aquatica, R. hydrolapathum E. angustifolium, C. rostrata and A. odoratum*) transported a higher proportion of CH_4_ internally (higher chimney effect) when the water level was high (Figure 1). However, two species, *L. europaeus,* and *G. maxima*, showed greater internal transport when the water level was low. These differences might reflect the differences among species in depth and distribution of roots in the soil [28]. For example, plants with a large proportion of active roots in the topsoil layer may only be able to transport significant proportions of CH_4_ when the water table is high, whereas plants with a deeper root system may also do so with a lower water table. These latter species could be expected to increase CH_4_ emissions from wetlands because they would conduct CH_4_ from deeper layers to the atmosphere, thereby by-passing the aerobic soil layer. However, we do not have empirical data on rooting patterns of different species to support this. If the difference in water table had been greater, the plant species might have behaved differently. Under field conditions, both the water table and rooting pattern of various species vary greatly, both spatially as well as temporally. To understand the relationship between variation of water table and transport capabilities of various plant species, however, would require further studies made using a range of water table depths.

We found that the capability of a plant to act as chimney was higher in species with higher plant biomass and root volume, particularly under low water level treatment (Figure 2a-h). If the plant-mediated transport were to be the dominant controlling factor, CH_4_ emissions would be higher from the species with high biomass. Several studies reported higher CH_4_ emissions from soil under high biomass or vegetation cover than those under low biomass or cover [21,23,42]. However, we observed the contrary, just as we consistently did in our previous experiments [11,17,44]. Both in low as well as high water level treatments, the CH_4_ emission was negatively related to plant biomass (Figure 3a-h). Some other studies also found either a negative or no relationship between plant productivity and CH_4_ emission [26,43]. A positive relationship of internal transport with root volume and/or plant biomass could reflect the greater ability of large plants to take up CH_4_ and transport it to atmosphere. However, a negative relationship of overall CH_4_ emissions with plant biomass is probably explained by increasing rhizosphere oxidation, since the amount of radial oxygen loss (ROL) from plant roots is known to depend on plant parameters such as leaf area, shoot diameter [54] and photosynthetic activity [55,56]. Presence of an extensive root system – besides contributing towards a higher chimney effect – may also enhance the oxidation of CH_4_ in the rhizosphere before it escapes to the atmosphere. This argument is supported by our results, showing that species with a high capacity to transport CH_4_ internally caused relatively lower emissions (Figure 4a-b). In a recent study comparing 35 wetland plant species, Lai *et al.*[56] found that ROL was positively related to the biomass of fine roots (diameter ≤ 1 mm), whereas it was negatively related to the biomass of thicker roots (diameter ≥ 3 mm). This suggests that characterisation of plant species based on root structure may form a basis for estimation of CH_4_ emissions from various vegetation zones.

These results imply that the plant species capable of transporting higher proportions of CH_4_ from rhizosphere to atmosphere also possess higher capacity to generate oxidising conditions in the rhizosphere [48,55]. The net balance of CH_4_ emission would be affected by the mechanism that dominates in a particular condition depending upon various abiotic factors (such as water table) and plant parameters (e.g. distribution and structure of root system). For example, we found that proportion of CH_4_ flux transported via plants (chimney effect) only correlated with plant biomass and root volume in the low water treatment (Figure 2). In a field study conducted in polygonal tundra in Siberia, it was found that vascular plants had a greater effect on CH_4_ emissions by enhancing CH_4_ oxidation at the elevated polygon-rim (water table 35–39 cm below soil surface), whereas in the polygon-centre (water table 0–4.5 cm below soil surface) CH_4_ transport and root exudation were dominant mechanisms [48].

## Conclusions

In conclusion, plants vary in their effects upon CH_4_ emissions from wetland soils. Species producing a large root volume and a relatively high biomass tend to transport proportionately more CH_4_ internally than species producing a smaller root volume. However, higher internal transport does not necessarily lead to higher CH_4_ emissions, as such plants may also favour the oxidation of CH_4_ in the rhizosphere. Since graminoids caused higher emissions, a shift in species composition from forbs to graminoids could lead to increased CH_4_ emissions. Conversely, a shift from low to high productivity may also result in lower CH_4_ emissions, as we found that overall CH_4_ emissions were negatively related to plant biomass. Future work should aim to produce a more quantitative understanding of how plants affect CH_4_ emissions based upon plant functional traits such as aerenchyma formation and root system features, particularly depth and distribution of roots and proportion of fine and coarse roots.

## Competing interests

The authors declare that they have no competing interests.

## Authors’ contributions

GSB, PJE and HOV designed the study; GSB and MI conducted the experiment and analysed the data. GSB prepared the first draft of manuscript and all authors revised and approved the final manuscript.
